# Spatially destabilising effect of woody plant diversity on forest productivity in a subtropical mountain forest

**DOI:** 10.1038/s41598-017-09922-7

**Published:** 2017-08-25

**Authors:** Yonglin Zhong, Yudan Sun, Mingfeng Xu, Yi Zhang, Yongqiang Wang, Zhiyao Su

**Affiliations:** College of Forestry and Landscape Architecture, South China Agricultural University, Guangzhou, 510642 China

## Abstract

We used geographically weighted regression to investigate the relationship between biodiversity and the spatial stability of forest productivity (SSFP) in a subtropical mountain forest. We examined the effect of elevation on this relationship and on its spatial non-stationarity. We found that higher woody plant diversity reduced SSPF. Higher woody plant diversity strengthened the asynchrony of species responses to spatial heterogeneity of forest habitats, which contributed to SSFP, but reduced two factors that enhanced SSFP: species dominance and the spatial stability of the dominant species. The percentage of variation in SSFP explained by diversity measures was highest for the Shannon-Wiener index, lowest for functional dispersion, and intermediate for species richness. The correlations of woody plant diversity with SSFP became stronger with elevation and varied among plots, indicating that the spatial non-stationarity existed in the biodiversity-SSFP relationship. These correlations became weaker in most cases after controlling for elevation. Our results suggest that in the subtropical mountain forest higher woody plant diversity has a spatially destabilising effect on forest productivity, particularly at higher elevations.

## Introduction

The accelerating loss of biodiversity and the degradation of ecosystem functions represent increasing threats to humans. Understanding the biodiversity-ecosystem functioning relationship is thus crucial for maintaining the delivery of ecosystem services on which humans depend^1–4^. As one of the most fundamental ecosystem functions^5^, stability refers to the resistance to change or disturbance to ecosystem structure and function across space and time^6–9^. Ecosystems with high stability can maintain a dynamic equilibrium of structure and consequently can guarantee the delivery of functions or services when faced with disturbances, especially when faced with environmental deterioration caused by anthropogenic activities^10, 11^. As an important aspect of ecosystem stability, temporal stability of ecosystem productivity (TSEP) is defined as the ratio of the mean value to the standard deviation of productivity across time and is strongly influenced by biodiversity^12, 13^. Mounting evidence has shown that greater biodiversity enhances the TSEP^14, 15^. Analogous to TSEP, spatial stability of ecosystem productivity is another important aspect of ecosystem stability, which reflects the response of ecosystem productivity to spatially environmental heterogeneity^7, 10^. However, whether biodiversity also enhances the spatial stability of ecosystem productivity remains unclear, particularly in forest ecosystems^7, 10^. Forests are major terrestrial ecosystems, and the stability of forest productivity plays an essential role in maintaining global biodiversity, as well as in stabilising the productivity of the global terrestrial ecosystems^11^. If biodiversity is positively correlated with the spatial stability of forest productivity (SSFP), then humans’ effort to maintain the terrestrial biodiversity^7^ will simultaneously contribute to SSFP.

Some ecologists believe that the effects of biodiversity on SSFP should be similar to its effects on TSEP^7, 10^. To explain the stabilising effect of biodiversity on TSEP, ecologists have proposed a variety of theories, including the overyielding effect, the portfolio effect, and species asynchrony^15–17^. Species asynchrony is regarded as one of the most important driving forces of TSEP. Plant species show different preferences for environmental conditions because of niche partitioning^18^. When a species-rich community experiences a disturbance, species that maximize fitness to the changed habitat will benefit, while others that are not adaptable to the new environment will be inhibited. Therefore, the overall productivity of a species-rich community will respond more stably to fluctuating environmental conditions than a species-poor community^12, 15, 17, 19^. Biodiversity may also affect TSEP through species dominance and the stability of the dominant species. A higher relative abundance of the most abundant species decreases the standard deviation relative to the mean value of productivity. Thus, species dominance is positively correlated with community stability^5^. A dominant species contributes most to community productivity. The stability of the dominant species plays an essential role in community stability^20^. Previous studies have demonstrated that higher biodiversity may lead to lower population stability^16^ and lower stability of the dominant species may destabilise community productivity^20^. However, we know little of how species asynchrony, species dominance, and the stability of the dominant species affect the relationship between biodiversity and SSFP^7, 10^.

Although most studies dealing with the effect of biodiversity on the stability of ecosystem productivity have used species richness as a diversity measure, species richness alone fails to account for the special roles of common and rare species in the community, the evenness of species distribution, species coexistence, and species-habitat associations^13, 21, 22^. In contrast, the Shannon-Wiener index involves both abundance and richness as predictors to quantify community heterogeneity^23, 24^. Similarly, the functional dispersion index considers not only richness but also niche complementarity of coexisting species and the functional traits of individual plant species^25, 26^. These diversity measures will presumably differ in their ability to explain the variations in the stability of ecosystems.

Both plant diversity and the stability in productivity are affected by temperature, moisture, and soil nutrients^27, 28^, which are in turn strongly influenced by elevation^29^. Soil nutrient content and availability change with temperature and precipitation^30, 31^. As elevation increases, temperature linearly decreases, whereas precipitation exhibits various patterns, such as linear, unimodal, or bimodal trends^32, 33^. Therefore, the biodiversity-stability relationship may also change with elevation-driven changes in environmental factors. Understanding the complex associations among plant diversity, SSFP, and the influence of elevation is crucial to the management and conservation of forests, especially mountain forests, and should also provide insight into the relationship between biodiversity and ecosystem functioning.

Here, we used geographically weighted regression (GWR) to investigate the relationship between biodiversity and SSFP, based on the data collected from 129 mountain forest plots established in the eastern part of Guangzhou. We used species richness, the Shannon-Wiener index, and functional dispersion as diversity measures, total tree basal area as a proxy variable for forest productivity, and the reciprocal of the coefficient of variation in the total tree basal areas among subplots within a plot as a proxy variable for SSFP. We also used Berger-Parker index to measure species dominance, and the reciprocal of the coefficient of variation in the tree basal areas of the dominant species among subplots within a plot as a proxy variable for spatial stability of the dominant species. We attempted to answer the following questions: (1) How are woody plant diversity, species asynchrony, the stability of the dominant species, and species dominance related to SSFP? (2) How do different diversity measures differ in explaining variations in SSFP? and (3) Does elevation affect the biodiversity-SSFP relationship? If so, how?

## Materials and Methods

### Study area

This study was conducted in the eastern part of Guangzhou (112°57′ - 114°3′ E, 22°26′ - 23°56′ N), which is located in the central south of Guangdong Province, China. Guangzhou has a subtropical monsoon climate regime and is consequently hot and wet in summer and cold and dry in winter, and is occasionally affected by typhoons and thunderstorms. The mean annual temperature and precipitation are 21.5 °C and 1667.5 mm, respectively. The wet season is from April to September. Most of the area has a south subtropical lateritic red soil whose parent rock is granite and sand shales^34^. The forests we investigated were evergreen broadleaved mountain forests with little disturbance. No signs of tree cutting and domestic animal grazing were observed in our monitoring plots.

### Data collection

To investigate the relationship between biodiversity and spatial stability of productivity in natural forest ecosystems, we established plots at 43 sites in the subtropical forests in eastern part of Guangzhou. At each site, we established three rectangular plots that were separated by at least 100 m. Each plot had an area of 1200 m^2^ (30 m × 40 m or 20 m × 60 m), and the total area of the 129 plots (43 sites × 3 plots/site) was 15.48 ha. Woody plant species richness in these plots ranged from 4 to 49. Each plot was further divided into 12 subplots of 10 m × 10 m.

We recorded the latitude, longitude, slope aspect, slope steepness, and elevation of each plot. Plot elevation ranged from 25 to 819 m a.s.l. The plots were grouped into four elevation classes for further analysis: 1 = 25–200 m; 2 = 201–400 m; 3 = 401–600 m; and 4 = 601–819 m. A tree census was conducted in each plot. We measured all woody plants with a diameter at breast height (DBH) ≥ 3 cm and labeled them with unique numbers. We recorded the species name, DBH (measured to the nearest 0.1 cm), and tree height (measured to the nearest 0.1 m) for all stems measured. All stems were identified to species during the survey (i.e., on site) except for those with uncertain identity; in the latter case, voucher specimens were collected, labeled, and subsequently identified by the South China Agricultural University Herbarium (CANT). Plant nomenclature follows Ye & Peng^35^. Field work for data collection was conducted and completed in 2014.

### Statistical analysis

We calculated species richness (*S*), the Shannon-Wiener index (*H*′), and functional dispersion (*FDis*) for plant diversity at the plot level using the following formulas:

1 $$S={\rm{number}}\,{\rm{of}}\,{\rm{species}}$$

2 $$H{\rm{\text{'}}}=-\sum _{{i}={1}}^{{S}}{piln}({pi})$$

3 $${FDis}=\sum _{{i}={1}}^{{S}}({aizi})/\sum _{{i}={1}}^{{S}}{ai}$$

where *p*_i_ is the relative individual density of species *i*; *a*_i_ is the individual density of species *i*, and *z*_i_ is the distance of species *i* to the weighted centroid c, which is determined by the individual density and functional trait value of each species^36^. Functional dispersion is based on maximum tree height, which is calculated as the 99^th^ percentile of the tree height values measured for each species.

Functional dispersion can be calculated based on any type and number of functional traits and any dissimilarity or distance measure, and is independent of the effect of species richness^36–38^. We used maximum tree height to calculate functional dispersion because height is associated with competition for light among trees and directly correlates with woody productivity^39^. In a few cases (18 in 25948 cases), we used multiple imputations to solve the problem of missing data in tree height values.

We used basal area as the proxy variable for forest productivity^40, 41^. SSFP was calculated as the ratio of the mean value to the standard deviation of productivity among subplots within each plot^7, 10^:

4 $${Stability}={\mu }/{\sigma }$$

Similarly, stability of the dominant species, the species with maximum total basal area at the plot level, was calculated as the ratio of the mean value to the standard deviation of productivity of the dominant species among subplots within each plot. We used the same formula that is used to calculate asynchronous responses of species across time^15, 28, 42^ in order to calculate asynchronic responses of species across space, but the meanings of the parameters changed:

5 $${Species}\,{Asynchrony}={1}-{{\sigma }}^{{2}}/{(\sum _{{i}={1}}^{{S}}{{\sigma }}_{{i}})}^{{2}}$$

where *σ*_*i*_ is the standard deviation of the productivity of species *i* among subplots within a plot, *σ* ^*2*^ is the variance of the productivity among subplots within a plot, and *S* is the species richness within a plot. Because some species within a plot occurred in only one or two subplots and because the calculation of standard deviation requires at least 3 replicates, we assumed that all species within a certain plot have individuals in each subplot of that plot. In cases where a species within a plot had no individual in any subplot of that plot, the productivity of that species in that subplot was recorded as zero. Asynchrony ranged from 0 to 1, where 0 indicated a perfectly synchronic response and 1 indicated a perfectly asynchronic response among different species to spatial heterogeneity in habitat. We calculated the Berger-Parker index to measure species dominance:

6 $${D}_{B-P}={N}_{\max }/N$$

where *D*_*B-P*_ is the Berger-Parker index, *N*_*max*_ is the number of individuals of the most abundant species, and *N* is the total number of individuals within each plot. Before conducting further analyses, we tested the normality of variables, and those violating the normality assumption were log_10_-transformed.

Although mounting evidence has demonstrated positive correlations between biodiversity and stability, the correlations and significance has differed among studies^12, 14, 15^. In one study, the biodiversity-stability relationships were even inconsistent among different sites^19^. These findings indicate spatial non-stationarity in correlations between biodiversity and temporal stability. Because the plots in our study were located in different areas of the eastern part of Guangzhou, we used geographically weighted regression (GWR) modeling to assess the relationship between woody plant diversity and SSFP because GWR models can efficiently deal with spatial non-stationarity^43, 44^. Model performance was assessed using the corrected Akaike Information Criterion (AICc), residual sum of squares (RSS), and the coefficient of determination (R^2^). Lower AICc and RSS values and higher R^2^ values indicate better model performance^44, 45^.

The effect of elevation was assessed by using the Kruskal-Wallis test to examine the significance of variation, along an elevational gradient, in the correlations of woody diversity with SSFP and of species asynchrony, species dominance, and stability of the dominant species with SSFP or woody plant diversity. After controlling for elevation, we used GWR models to evaluate the relationship between woody plant diversity and SSFP; elevation was controlled for by using the residuals of SSFP on elevation^45^. Correlations of species asynchrony, species dominance, and stability of the dominant species with plant diversity or SSFP after controlling for elevation were examined using the same methods.

R software version 3.2.5^46^ was used for all calculations and statistical analyses. Multiple imputations were conducted using the *mice* package^47^. The Shannon-Wiener index and functional dispersion were calculated using *vegan* ^48^ and *FD* ^36^ packages, respectively. Normality tests and Kruskal-Wallis tests were performed using the *stats* package^46^. GWR analyses were conducted with the *spgwr* package^49^, using a Gaussian spatial weighting function with an adaptive spatial kernel.

## Results

### Plant diversity-SSFP relationship

**Figure 1:**
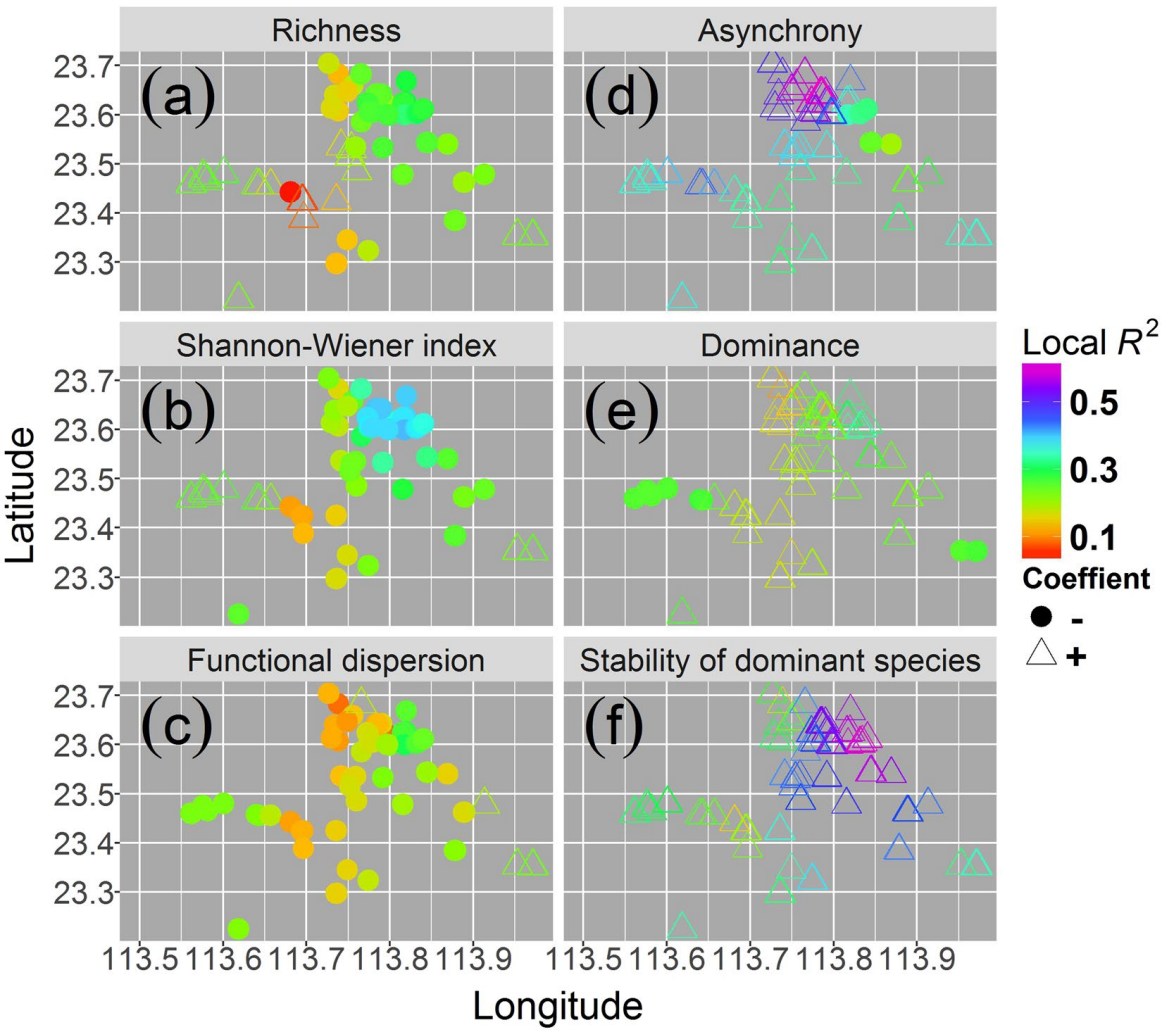
Local R^2^ for geographically weighted regressions of SSFP against various predictive variables. The predictive variables: (**a**) species richness, (**b**) the Shannon-Wiener index, (**c**) functional dispersion, (**d**) species asynchrony, (**e**) species dominance, and (**f**) stability of the dominant species. Filled circles represent negative correlations, and triangles represent positive correlations.

Although the three measures of woody plant diversity explained significant proportions of the variances of SSFP (overall R^2^ > 0.20, Table 1), the measures differed in explanatory power. The Shannon-Wiener index explained the most variance (overall R^2^ = 0.280), while functional dispersion explained the least (overall R^2^ = 0.219). According to the AICc and RSS values, the GWR model for SSFP against Shannon-Wiener index was fitted better than those against species richness or functional dispersion (Table 1). Spatial non-stationarity existed in the correlations between SSFP and plant diversity. SSFP was negatively correlated with plant diversity in most plots and was positively correlated with plant diversity in only a few plots. Local R^2^ of GWR models for SSFP against plant diversity varied strongly among plots (Fig. 1a–c). Correlations between SSFP and species richness or the Shannon-Wiener index were highly consistent in both magnitude and direction among plots (Fig. 1a,b). Correlations between SSFP and species diversity (species richness or Shannon-Wiener index) showed larger ranges of variations among plots than the correlations between SSFP and functional diversity (functional dispersion) (Fig. 1a–c).

**Table 1:** Results from geographically weighted regressions of spatial stability of forest productivity (SSFP), species asynchrony, species dominance, and the stability of dominant species, respectively, as the response variable against various predictive variables.

Response vs. predictive variables	Without controlling for elevation			After controlling for elevation		
	AICc	R^2^	RSS	AICc	R^2^	RSS
SSFP vs.						
Species richness	−123.187	0.242	2.368	−132.959	0.124	2.195
Shannon-Wiener index	−129.348	0.280	2.248	−138.900	0.166	2.088
Functional dispersion	−117.715	0.219	2.440	−130.895	0.120	2.203
Species asynchrony	−152.389	0.397	1.884	−167.834	0.333	1.671
Species dominance	−124.567	0.260	2.312	−152.940	0.158	2.109
Stability of dominant species	−154.773	0.408	1.848	−167.201	0.330	1.679
Species asynchrony vs.						
Species richness	−338.291	0.523	0.447	−350.429	0.314	0.407
Shannon-Wiener index	−370.161	0.629	0.348	−375.898	0.440	0.333
Functional dispersion	−301.644	0.375	0.586	−331.524	0.216	0.465
Species dominance vs.						
Species richness	−92.731	0.523	2.999	−103.048	0.346	2.768
Shannon-Wiener index	−203.773	0.799	1.263	−197.867	0.688	1.322
Functional dispersion	−78.565	0.474	3.306	−113.492	0.404	2.522
Stability of dominant species vs.						
Species richness	−23.005	0.250	5.149	−30.809	0.114	4.846
Shannon-Wiener index	−51.149	0.399	4.122	−52.498	0.254	4.079
Functional dispersion	−17.159	0.225	5.321	−31.279	0.128	4.769

All variables except functional dispersion were log_10_-transformed before analyses. Abbreviations: AICc = the corrected Akaike Information Criterion; R^2^ = the global coefficient of determination; RSS = residual sum of squares.

### Explanatory power of predictors for the plant diversity-SSFP relationship

**Figure 2:**
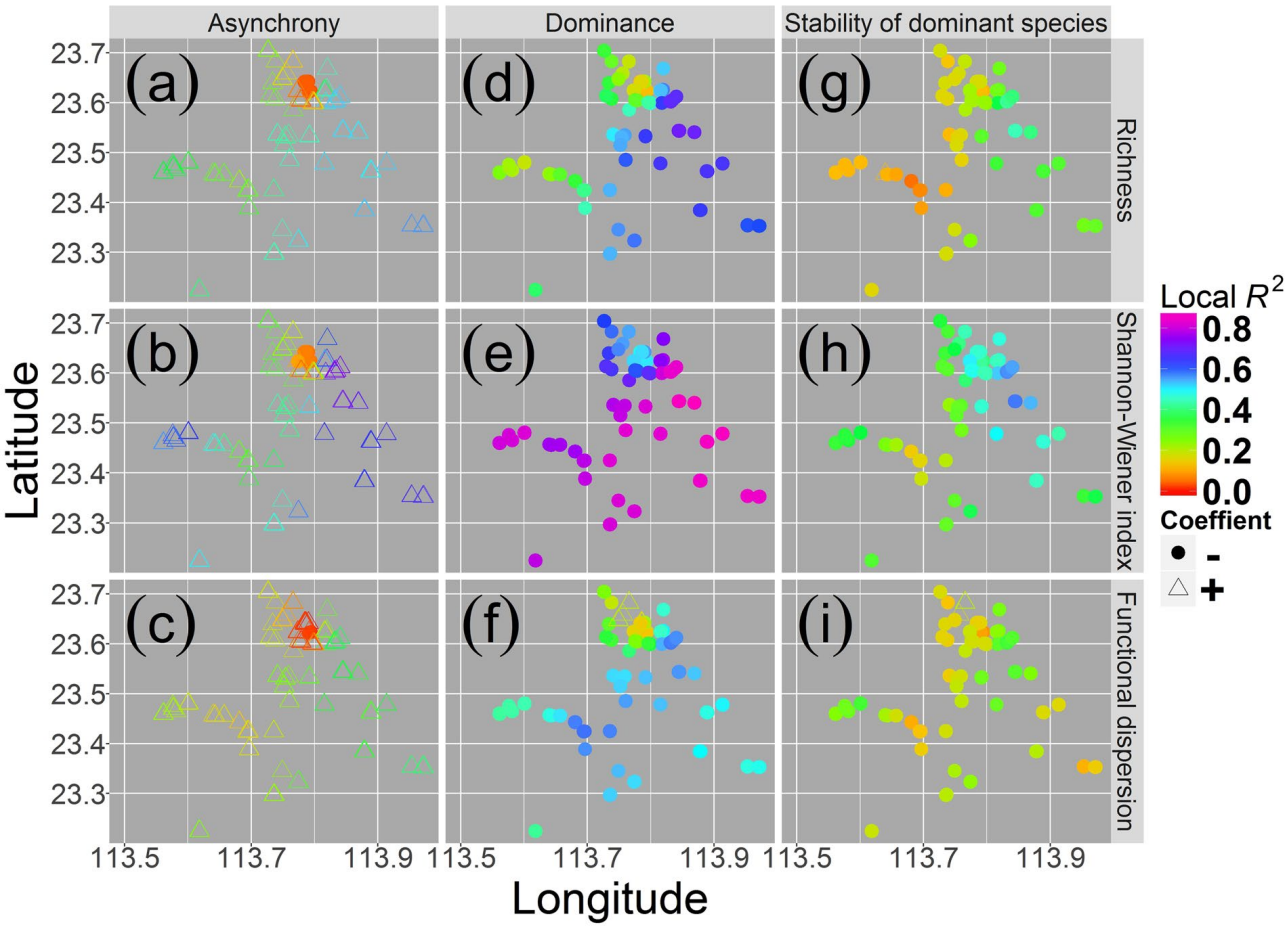
Local R^2^ for geographically weighted regressions of species asynchrony, species dominance, and stability of the dominant species, respectively, against diversity measures. 1) species asynchrony against (**a**) species richness, (**b**) Shannon-Wiener index, and (**c**) functional dispersion; 2) species dominance against (**d**) species richness, (**e**) the Shannon-Wiener index, and (**f**) functional dispersion; 3) stability of the dominant species against (**g**) species richness, (**h**) the Shannon-Wiener index, and functional dispersion. Filled circles represent negative correlations, and triangles represent positive correlations.

GWR model for SSFP against stability of the dominant species was fitted better than those against species asynchrony or species dominance. As assessed by AICc and RSS values, GWR models for species asynchrony, species dominance, or stability of the dominant species against Shannon-Wiener index were fitted better than those against species richness or functional dispersion (Table 1). Overall, more of the variance in SSFP was explained by species asynchrony, species dominance, or stability of the dominant species than by plant diversity measures. Among species asynchrony, species dominance, and stability of the dominant species, stability of the dominant species explained the most variance (overall R^2^ = 0.408), and species dominance explained the least variance (overall R^2^ = 0.260) in SSFP. Plant diversity had a strong explanatory power for variances of species asynchrony, species dominance, and stability of the dominant species. Similarly, the Shannon-Wiener index explained the most variance and functional dispersion explained the least variance in species asynchrony, species dominance, and stability of the dominant species (Table 1). Spatial non-stationarity also existed in correlations of species asynchrony, species dominance, or stability of the dominant species with SSFP and with plant diversity measures. These correlations varied strongly among plots (Figs 1 and 2). SSFP was positively correlated with species asynchrony and species dominance in most plots and was negatively correlated with these variables in only a few plots (Fig. 1d,e). SSFP was positively correlated with stability of the dominant species in all plots (Fig. 1f). Species asynchrony was positively correlated with plant diversity in most plots and was negatively correlated with plant diversity in only a few plots (Fig. 2a–c). Both species dominance and stability of the dominant species were negatively correlated with plant diversity in most plots and were positively correlated with plant diversity in only a few plots (Fig. 2d–i). Species dominance was negatively correlated with species diversity in all plots. Stability of the dominant species was also negatively correlated with the Shannon-Wiener index in all plots (Fig. 2d,e,h).

### Influence of elevation

**Figure 3:**
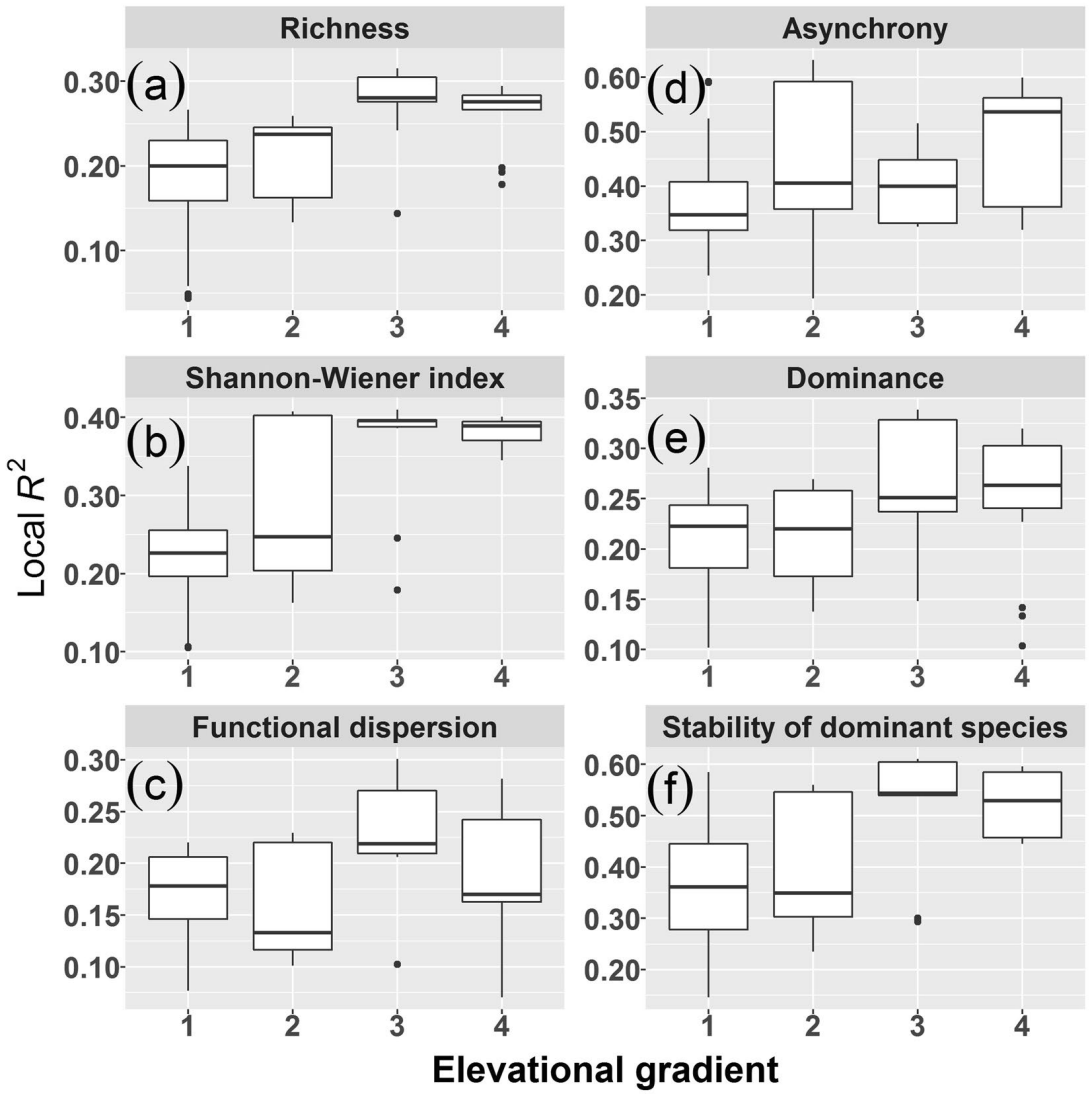
Effects of elevation on the local *R* ^2^ for geographically weighted regression of SSFP against various predictive variables. The predictive variables: (**a**) species richness (KW-H(3, 129) = 39.789, *P* < 0.0001), (**b**) the Shannon-Wiener index (KW-H(3, 129) = 42.592, *P* < 0.0001), (**c**) functional dispersion (KW-H(3, 129) = 12.040, *P* = 0.0072), (**d**) species asynchrony (KW-H(3, 129) = 18.299, *P* = 0.0004), (**e**) species dominance (KW-H(3, 129) = 13.091, *P* = 0.0044), and (**f**) stability of the dominant species (KW-H(3, 129) = 29.666, *P* < 0.0001). Boxes represent the 25^th^ and 75^th^ percentile values, the horizontal line in the box is the median, the whiskers show the non-outlier range, and the solid points represent outliers. Differences along the elevation gradient were tested using Kruskal-Wallis H test. Elevation gradient: 1 = 0–200 m; 2 = 201–400 m; 3 = 401–600 m; 4 = 601–819 m.

Changes in elevation influenced the relationships of SSFP to plant diversity, species asynchrony, species dominance, and the stability of the dominant species. As elevation increased, the local R^2^ tended to increase significantly for regressions of SSFP against species diversity (*P* < 0.0001, Fig. 3a,b), species asynchrony (*P* = 0.0004, Fig. 3d), species dominance (*P* = 0.0044, Fig. 3e), and stability of the dominant species (*P* < 0.0001, Fig. 3f), but fluctuate significantly for the regression of SSFP against functional dispersion (*P* = 0.0072, Fig. 3c). The local R^2^ for regressions of species asynchrony, species dominance, and stability of the dominant species, respectively, against plant diversity varied, either significantly increased, significantly decreased, significantly fluctuated, or remained relatively constant with increasing elevation (see Supplementary Fig. S1).

After elevation was controlled for, the overall R^2^ decreased for all models, but the independent variables still explained more than 10% of the variances of the dependent variables (Table 1). Controlling for elevation did not change the direction of the correlations in most plots for any model, but decreased the magnitude of the variation in correlations, except for the correlations of SSFP with species asynchrony, for correlations of species dominance with Shannon-Wiener index or functional dispersion, or for the correlations of stability of the dominant species with functional dispersion; for these exceptions, controlling for elevation increased the magnitude of variations (Figs 1 and 2; Supplementary Figs S2 and S3).

## Discussion

Species with different preferences for certain habitats respond differently to environmental changes across time or space. Some species are enhanced while others are inhibited or unaffected by changes in the environment, reflecting different adaptations to the habitat changes. In addition, the speed at which organisms respond to environmental change differs among species. Communities with higher diversity contain more species with contrasting preferences for environmental changes, and this diversity therefore tends to increase community stability^15, 17^. This view is to some extent supported by the results of our study. We found that the SSFP was positively associated with species asynchrony across space, which in turn was positively associated with woody plant diversity. However, SSFP was actually reduced by plant diversity in most plots. This can be explained by our finding that both species dominance and the stability of the dominant species were positively correlated with SSFP but negatively correlated with plant diversity. The effects of species dominance and spatial stability of the dominant species overrode that of species asynchrony. These results suggest that plant diversity has more destabilising than stabilising effects on forest productivity across space and that species dominance and the stability of the dominant species, rather than species asynchrony, determine the relationship of plant diversity to SSFP. Higher species dominance increased temporal stability by lowering the standard deviation relative to the mean value of forest productivity across time, indicating that species dominance was positively correlated with temporal stability^5^. Species dominance was also positively correlated with SSFP (Fig. 1e). As plant diversity increased, species dominance decreased, consequently leading to lower SSFP. A dominant species occupies larger ecological niches, is more adaptable to environmental changes^50^, and is more stable than other species^20, 51^. Furthermore, the most dominant species is the major contributor of total community productivity and thus contributes most to the stability of forest productivity. Changes in the stability of the dominant species will affect temporal stability of ecosystem productivity^20^. Higher plant diversity leads to lower population stability, particularly the stability of both the dominant and rare species, because of competition^16^, and decreases in the stability of the dominant species destabilise community productivity across time^20^. In our study, higher plant diversity also reduced SSFP by decreasing the spatial stability of the dominant species. We therefore regarded the decrease in species dominance and in the stability of the dominant species with the increase in plant diversity as key factors explaining the destabilising effect of diversity on SSFP. We also found that the Shannon-Wiener index explained more of the variance in the diversity-SSFP relationship than the other diversity measures used in this study. This might be because the Shannon-Wiener index combines the richness, abundance, and evenness of species, and thereby considers the special roles of the dominant, common, and rare species in the community^24^, and because the dominant species greatly affects community stability^5, 52^.

The responses of spatial stability of productivity to biodiversity can differ among ecosystems. In contrast to the results from our forest study, results of the Jena grassland study suggested that biodiversity is positively correlated with spatial stability of productivity and that only functional trait diversity can explain the variations in spatial stability^10^. Previous studies have demonstrated that woody plants are more sensitive to environmental change than grasses, which might explain the discrepancy in the relationship between biodiversity and spatial stability in forest versus grassland ecosystems^53, 54^.

We found that the correlation of woody plant diversity with SSFP and correlations of species asynchrony, species dominance, and stability of the dominant species with plant diversity and with SSFP were affected by elevation. As temperature linearly decreases and precipitation distribution patterns change with increasing elevation^32, 33^, soil moisture and nutrients also change^30, 31^. Changes in these environmental factors can drive variations in woody plant diversity, productivity, species asynchrony, species dominance, and the stability of the dominant species in a forest ecosystem, thus leading to variations in SSFP^20, 27, 28, 42, 55^. As elevation increased in the current study, the effects of species diversity on SSFP significantly increased and quickly saturated at higher elevations. These findings suggest that higher woody plant diversity, particularly species diversity, reduced the stability of forest productivity across space in higher-elevation regions and that the destabilising effect of plant diversity on forest productivity across space might be stronger at higher elevations. That species asynchrony, species dominance, and the stability of the dominant species had stronger associations with SSFP as elevation increased demonstrated that their stabilising effects on SSFP became stronger as elevation increased. The different rates at which these correlations (coefficients of determination) increased with elevation demonstrated that the relative importance of species asynchrony to species dominance and the stability of the dominant species fluctuated as elevation increased. Correlations between plant diversity and SSFP, and correlations of species asynchrony, species dominance, and the stability of the dominant species with SSFP and plant diversity decreased when elevation was controlled for, demonstrating that elevation had a strong effect on the SSFP, and that the effect of elevation might be even stronger than that of plant diversity.

Spatial non-stationarity was evident in the diversity-SSFP relationship and decreased in most cases when elevation was controlled for, indicating that elevation affects but does not determine the spatial non-stationarity in the correlations of diversity with SSFP. Environmental heterogeneity and the interactions between species and environment are regarded as two important drivers of spatial non-stationarity in the diversity-stability relationship^15^. The growth, development, and distribution of plants are associated with habitat heterogeneity^54, 56, 57^, which is controlled by elevation-driven variations in temperature, moisture, and soil nutrients^30–33^. Thus, both plant diversity and ecosystem stability, and consequently their correlations, are affected by elevation-driven changes in a habitat^32, 42, 55^. However, habitat variations are driven by many biotic and abiotic factors, including slope aspect, slope steepness, anthropogenic disturbance, and biological invasion^58–61^. Further studies are therefore needed to determine how diversity affects SSFP and how the relationship between diversity and SSFP is influenced by spatial non-stationarity at multiple spatial scales.

In conclusion, our results demonstrated that plant diversity increased species asynchrony, which in turn promoted SSFP; however, due to its inverse association with species dominance and the stability of the dominant species, which are the major drivers of SSFP, higher plant diversity reduced SSFP. The destabilising effect of plant diversity on SSFP became stronger with elevation, indicating that higher plant diversity further reduced the SSFP at higher elevations. The proportion of variation in SSFP explained by diversity measures was highest for the Shannon-Wiener index, lowest for functional dispersion, and intermediate for species richness. These results will help expand our understanding of the biodiversity-ecosystem functioning relationship and will have potential implications for biodiversity conservation and forest management.

### Data Availability

The datasets generated during the current study are available from the corresponding author on reasonable request.

## Electronic supplementary material

Supplementary Figures
