# Supplementary material for: Spatially destabilising effect of woody plant diversity on forest productivity in a subtropical mountain forest
Source: Sci Rep. 2017 Aug 25;7:9551. doi: 10.1038/s41598-017-09922-7 (PMC5573360; doi:10.1038/s41598-017-09922-7)
Supplement: Supplementary file 1 — Supplementary Figures [file 41598_2017_9922_MOESM1_ESM.pdf]

Spatially destabilizing effect of woody plant diversity on forest productivity in a subtropical mountain forest

Yonglin Zhong, Yudan Sun, Mingfeng Xu, Yi Zhang, Yongqiang Wang, Zhiyao Su*

College of Forestry and Landscape Architecture, South China Agricultural University, Guangzhou 510642, China

*Corresponding author: zysu@scau.edu.cn

Supplementary Information

1. Supplementary Figures

Figure S1, Figure S2, and Figure S3

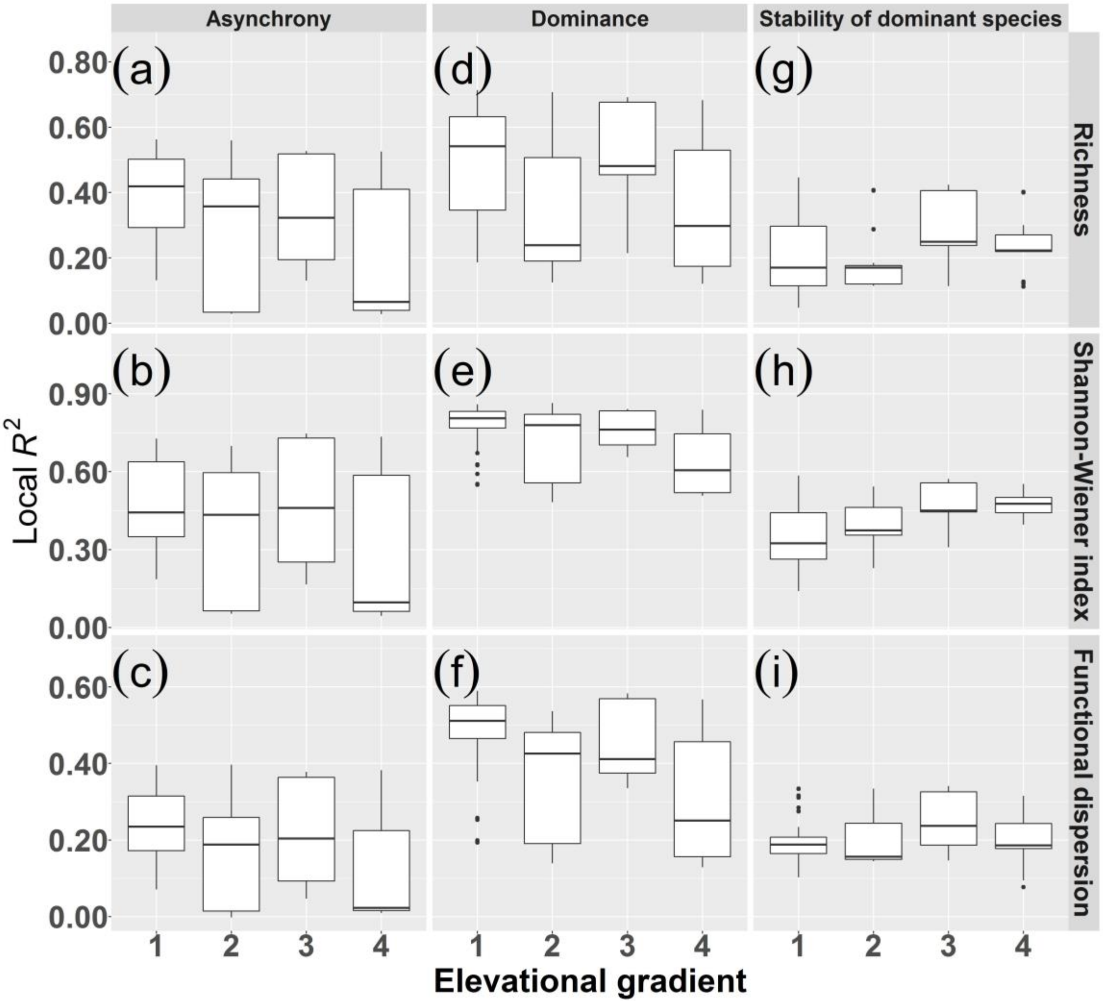

Supplementary Figure S1. Effects of elevation on the local R^2 for geographically weighted regression of species asynchrony, species dominance, and stability of the dominant species, respectively, against diversity measures. 1) species asynchrony against (a) species richness (KW-H(3, 129) = 13.671, $P = 0.0034$), (b) the Shannon-Wiener index (KW-H(3, 129) = 7.555, $P = 0.0562$), and (c) functional dispersion (KW-H(3, 129) = 11.592, $P = 0.0089$); 2) species dominance against (d) species richness (KW-H(3, 129) = 18.184, $P = 0.0004$), (e) the Shannon-Wiener index (KW-H(3, 129) = 18.268, $P = 0.0004$), and (f) functional dispersion (KW-H(3, 129) = 27.453, $P < 0.0001$); 3) stability of the dominant species against (g) species richness (KW-H(3, 129) = 9.865, $P = 0.0198$), (h) the Shannon-Wiener index (KW-H(3, 129) = 29.956, $P < 0.0001$), and functional dispersion (KW-H(3, 129) = 6.536, $P = 0.0882$). Boxes represent the 25th and 75th percentile values, the horizontal line in the box is the median, the whiskers show the non-outlier range, and the solid points represent outliers. Differences along the elevation gradient were tested using Kruskal-Wallis H test. Elevation gradient: 1 = 0–200 m; 2 = 201–400 m; 3 = 401–600 m; 4 = 601–819 m.

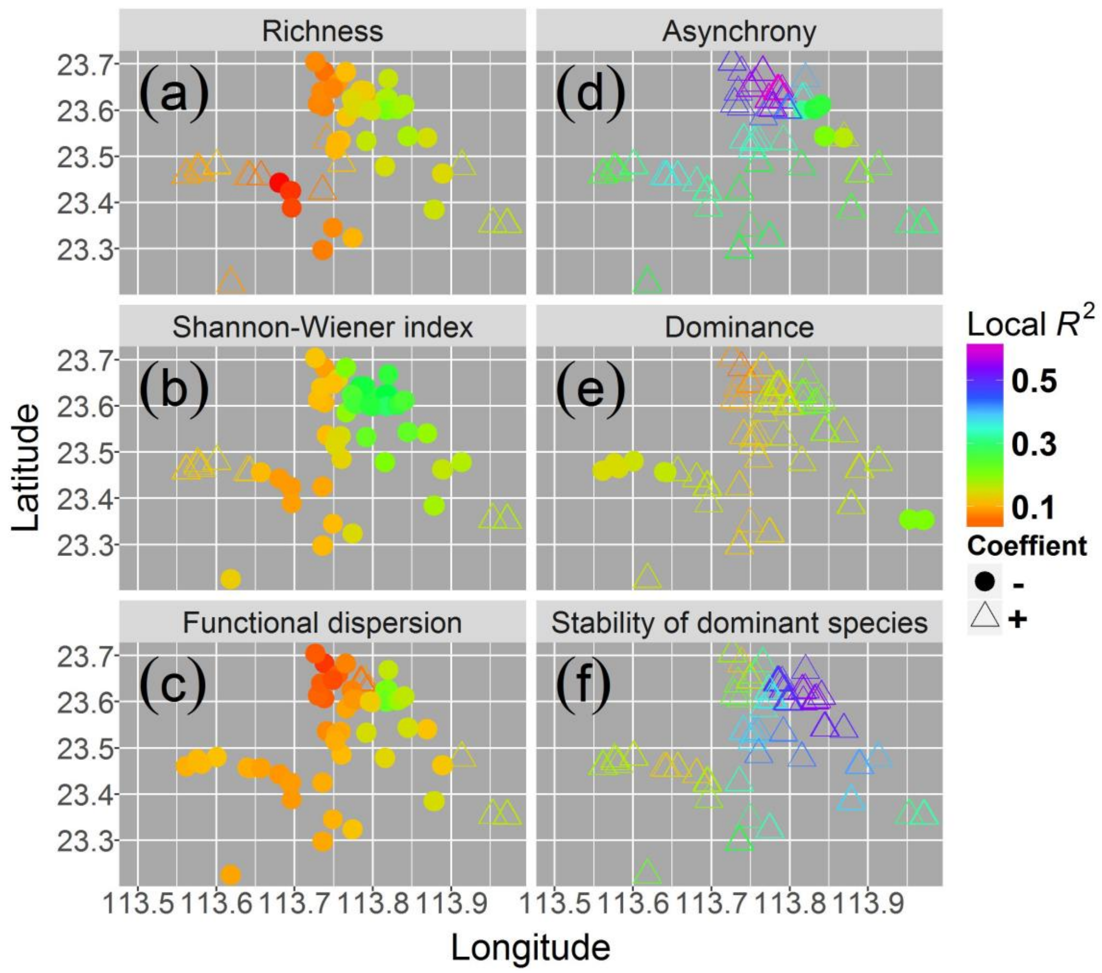

Supplementary Figure S2. Local R^2 for geographically weighted regressions of SSEP against various predictive variables, after controlling for elevation. The predictive variables: (a) species richness, (b) the Shannon-Wiener index, (c) functional dispersion, (d) species asynchrony, (e) species dominance, and (f) stability of the dominant species. Filled circles represent negative correlations, and triangles represent positive correlations.

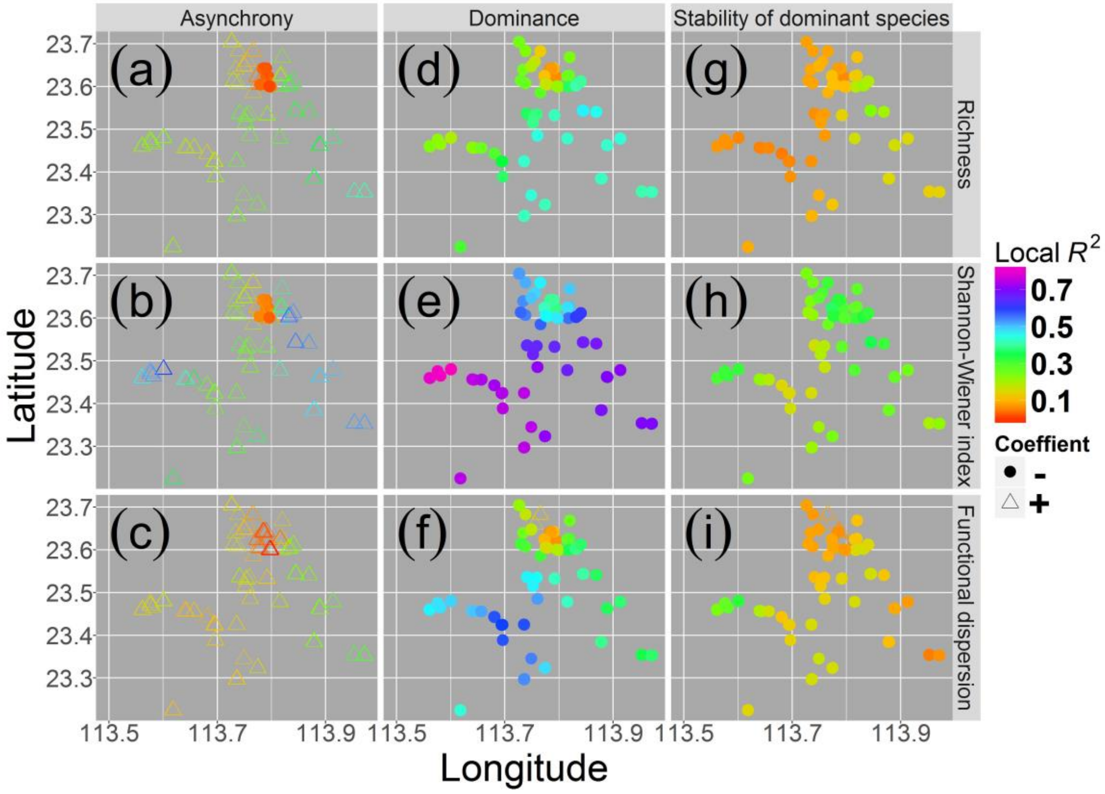

Supplementary Figure S3. Local R^2 for geographically weighted regressions of species asynchrony, species dominance, and stability of the dominance species, respectively, against diversity measures, after controlling for elevation. 1) species asynchrony against (a) species richness, (b) the Shannon-Wiener index, and (c) functional dispersion; 2) species dominance against (d) species richness, (e) the Shannon-Wiener index, and (f) functional dispersion; 3) stability of the dominant species against (g) species richness, (h) the Shannon-Wiener index, and functional dispersion. Filled circles represent negative correlations, and filled triangles represent positive correlations.
